# Supplementary material for: Natural depletion of histone H1 in sex cells causes DNA demethylation, heterochromatin decondensation and transposon activation
Source: eLife. 2019 May 28;8:e42530. doi: 10.7554/eLife.42530 (PMC6594752; doi:10.7554/eLife.42530)
Supplement: Transparent reporting form [file elife-42530-transrepform.docx]

***eLife’s* transparent reporting form**

We encourage authors to provide detailed information *within their submission* to facilitate the interpretation and replication of experiments. Authors can upload supporting documentation to indicate the use of appropriate reporting guidelines for health-related research (see [EQUATOR Network](http://www.equator-network.org/%20)), life science research (see the [BioSharing Information Resource](https://biosharing.org/)), or the [ARRIVE guidelines](http://www.plosbiology.org/article/info:doi/10.1371/journal.pbio.1000412) for reporting work involving animal research. Where applicable, authors should refer to any relevant reporting standards documents in this form.

If you have any questions, please consult our Journal Policies and/or contact us: [editorial@elifesciences.org](mailto:editorial@elifesciences.org).

**Sample-size estimation**

- You should state whether an appropriate sample size was computed when the study was being designed
- You should state the statistical method of sample size computation and any required assumptions
- If no explicit power analysis was used, you should describe how you decided what sample (replicate) size (number) to use

Please outline where this information can be found within the submission (e.g., sections or figure legends), or explain why this information doesn’t apply to your submission:

1. Three biological replicates were used for RNA-seq and qRT-PCR for each genotype; info can be found in the Results section, the legend of Figure 3, and the Materials and Methods.
2. More than 100 nuclei or late microspores were imaged for each genotype in Figure 5; info can be found in the figure panel.

**Replicates**

- You should report how often each experiment was performed
- You should include a definition of biological versus technical replication
- The data obtained should be provided and sufficient information should be provided to indicate the number of independent biological and/or technical replicates
- If you encountered any outliers, you should describe how these were handled
- Criteria for exclusion/inclusion of data should be clearly stated
- High-throughput sequence data should be uploaded before submission, with a private link for reviewers provided (these are available from both GEO and ArrayExpress)

Please outline where this information can be found within the submission (e.g., sections or figure legends), or explain why this information doesn’t apply to your submission:

1. Three biological replicates were used for each genotype for pollen RNA-seq and qRT-PCR (described in the Materials and Methods). For each biological replicate, pollen grains collected from flowers of different plants grown under the same condition were used for RNA extraction.
2. In the Author Information, GEO accession number for high-throughput sequencing data was provided. The reviewer token to access the data is provided in the cover letter.

**Statistical reporting**

- Statistical analysis methods should be described and justified
- Raw data should be presented in figures whenever informative to do so (typically when N per group is less than 10)
- For each experiment, you should identify the statistical tests used, exact values of N, definitions of center, methods of multiple test correction, and dispersion and precision measures (e.g., mean, median, SD, SEM, confidence intervals; and, for the major substantive results, a measure of effect size (e.g., Pearson's r, Cohen's d)
- Report exact p-values wherever possible alongside the summary statistics and 95% confidence intervals. These should be reported for all key questions and not only when the p-value is less than 0.05.

Please outline where this information can be found within the submission (e.g., sections or figure legends), or explain why this information doesn’t apply to your submission:

1. In Results section, the legend of Figure 3, and the Materials and Methods, Sleuth program was used with the default settings including likelihood ratio test to identify VC-expressed TEs and H1-repressed TEs, respectively.
2. In Results section, Fisher’s exact test was done to identify TE superfamilies enriched in VC-activated or H1-repressed TEs; to identify H1 hyperDMRs that are DME targets and vice versa; to identify TEs whose DNA methylation within 500bp of TSSs is increased by VC-expressed H1.
3. In the legends of Figure 1, Figure 2, Figure 3, and Figure 1—figure supplement 1, Kolmogorov-Smirnov test was done to show the differences between the indicated datasets in terms of various genomic features.
4. In the legend of Figure 3, Student’s t test was done to show the differences among samples.
5. In Materials and Methods section, Fisher’s exact test was done to identify VC DME targets; to identify the most demethylated cytosines by DME in VC; to identify TEs whose DNA methylation within 500bp of TSSs is increased by VC-expressed H1.

(For large datasets, or papers with a very large number of statistical tests, you may upload a single table file with tests, Ns, etc., with reference to sections in the manuscript.)

**Group allocation**

- Indicate how samples were allocated into experimental groups (in the case of clinical studies, please specify allocation to treatment method); if randomization was used, please also state if restricted randomization was applied
- Indicate if masking was used during group allocation, data collection and/or data analysis

Please outline where this information can be found within the submission (e.g., sections or figure legends), or explain why this information doesn’t apply to your submission:

No such information was involved in this study.

**Additional data files (“source data”)**

- We encourage you to upload relevant additional data files, such as numerical data that are represented as a graph in a figure, or as a summary table
- Where provided, these should be in the most useful format, and they can be uploaded as “Source data” files linked to a main figure or table
- Include model definition files including the full list of parameters used
- Include code used for data analysis (e.g., R, MatLab)
- Avoid stating that data files are “available upon request”

Please indicate the figures or tables for which source data files have been provided:

Two, one, and two source data files have been provided for Figure 1, Figure 2, and Figure 3, respectively.
